# Distribution of tuberculosis cases in the state of Paraná: an ecological study, Brazil, 2018-2021

**DOI:** 10.1590/S2237-96222023000200010

**Published:** 2023-06-19

**Authors:** Lucas Vinícius de Lima, Gabriel Pavinati, Andressa Aya Ohta, Nelly Lopes de Moraes Gil, Débora Regina de Oliveira Moura, Gabriela Tavares Magnabosco

**Affiliations:** 1 Universidade Estadual de Maringá, Programa de Pós-Graduação em Enfermagem, Maringá, PR, Brazil; 2 Universidade Estadual de Maringá, Departamento de Enfermagem, Maringá, PR, Brazil

**Keywords:** Pulmonary Tuberculosis, Epidemiology, Public Health, Spatial Analysis, Ecological Studies, Tuberculosis Pulmonar, Epidemiología, Salud Pública, Análisis Espacial, Estudios Ecológicos, Tuberculose Pulmonar, Epidemiologia, Saúde Pública, Análise Espacial, Estudos Ecológicos

## Abstract

**Objective:**

to analyze the distribution of tuberculosis cases in the state of Paraná, Brazil, between 2018 and 2021.

**Methods:**

this was an ecological study using secondary data obtained from compulsory notifications; detection rates per 100,000 inhabitants were described according to health regions in the state; percentage changes between 2018-2019 and 2020-2021 were calculated.

**Results:**

a total of 7,099 cases were registered. The highest rates were observed in the health regions of Paranaguá (52.4/100,000 in 2018-2019; 38.2/100,000 in 2020-2021) and Foz do Iguaçu (34.4/100,000 in 2018-2019; 20.5/100,000 in 2020-2021), and the lowest rates in Irati (6.3/100,000 in 2018-2019; 8.8/100,000 in 2020-2021) and Francisco Beltrão (8.5/100,000 in 2018-2019; 7.6/100,000 in 2020-2021); in 2020-2021, it could be seen a decrease in percentage changes in 18 health regions, while there was an increase in four of them, especially Foz do Iguaçu (-40.5%) and Cianorte (+53.6%).

**Conclusion:**

high rates were found in the coastal and triple border regions; and there was a decline in detection rates in the pandemic period.

## RESUMO

**Objetivo:**

analisar a distribuição dos casos de tuberculose no Paraná, Brasil, entre 2018 e 2021.

**Métodos:**

estudo ecológico, sobre dados secundários oriundos de notificações compulsórias; descrição das taxas de detecção por 100 mil habitantes segundo regiões de saúde do estado; cálculo das variações percentuais entre 2018-2019 e 2020-2021.

**Resultados:**

foram registrados 7.099 casos, observando-se maiores taxas nas regionais de Paranaguá (52,4/100 mil em 2018-2019; 38,2/100 mil em 2020-2021) e Foz do Iguaçu (34,4/100 mil em 2018-2019; 20,5/100 mil em 2020-2021), e menores em Irati (6,3/100 mil em 2018-2019; 8,8/100 mil em 2020-2021) e Francisco Beltrão (8,5/100 mil em 2018-2019; 7,6/100 mil em 2020-2021); em 2020-2021, houve queda nas variações percentuais dessas taxas em 18 regionais e aumento em quatro, destacando-se, respectivamente, Foz do Iguaçu (-40,5%) e Cianorte (+53,6%).

**Conclusão:**

foram observadas taxas elevadas nas regionais do litoral e da tríplice fronteira; houve declínio das taxas de detecção no período pandêmico.

## RESUMEN

**Objetivo:**

analizar la distribución de casos de tuberculosis en Paraná de 2018 a 2021.

**Métodos:**

estudio ecológico utilizando datos secundarios de notificaciones obligatorias. Se describieron las tasas de detección por 100.000 habitantes según las regiones de salud de Paraná. Se calcularon los cambios porcentuales entre 2018-2019 y 2020-2021.

**Resultados:**

se registraron 7.099 casos, observándose tasas más altas en las regiones de Paranaguá (52,4/100.000 en 2018-2019 y 38,2/100.000 en 2020-2021) y Foz do Iguaçu (34,4/100.000 en 2018-2019 y 20,5/100.000 en 2020-2021) y menor en Irati (6,3/100.000 en 2018-2019 y 8,8/100.000 en 2020-2021) y Francisco Beltrão (8,5/100.000 en 2018-2019 y 7,6/100.000 en 2020-2021). En 2020-2021, hubo descenso en 18 regiones y aumento en cuatro, con destaque para Foz do Iguaçu (-40,5%) y Cianorte (+53,6%).

**Conclusión:**

se encontraron altas tasas para las regiones de la costa y la triple frontera, además de una disminución en la detección durante el período de pandemia.

**Table:** 

Study contributions	
**Main results**	Between the biennia 2020-2021 and 2018-2019, there was a decrease in the detection of tuberculosis in 18 of the 22 health regions of Paraná. The cases were concentrated in the North and Northwest macro-regions and in the border and coastal regions of the state.
**Implications for services**	This scenario threatens the achievement in national targets to end tuberculosis as a public health problem, thus future control strategies aimed at preventing, early diagnosis, and effective treatment of the condition are necessary.
**Perspectives**	The results can support the targeting of effective actions aimed at the control of tuberculosis in health regions with higher occurrence, in addition to highlighting the need to strengthen surveillance and health care in a crisis context.

## INTRODUCTION

Tuberculosis (TB) is a major public health problem and one of the leading causes of deaths from infectious diseases worldwide.^1^ ^)^ Until the advent of the COVID-19 pandemic in 2020, when historical progress in controlling and coping with several health conditions was reversed, TB was the leading cause of death from a single infectious agent, ranking above acquired immunodeficiency syndrome (or AIDS, as the disease is more commonly known).^1^ ^)-(^ ^3^

In 2019, 7.1 million people were diagnosed with TB worldwide, followed by a decline in cases of the disease to approximately 5.8 million in 2020, far short the 10 million expected by the World Health Organization (WHO).^1^ Brazil and 15 other countries accounted for about 93% of this decline;^4^ in 2021, just over 68,000 cases were reported in the country, 2.7% of them in the state of Paraná.^4^

The End TB Strategy, a program launched by the WHO in 2015, has set ambitious targets of 90% reduction in TB incidence and 95% reduction in number of TB deaths by 2035.^5^ In Brazil, public health has agreed on recommendations formalized by Brazil Free from Tuberculosis: National Plan to End Tuberculosis as a Public Health Problem, a health action plan launched in 2017 and revised in 2021.^6^

Although successful results have been achieved on the track to end TB, it could be seen that the decline in notifications of cases is still insufficient to meet national and global targets,^3^ requiring further efforts to improve the capacity of surveillance systems, achieve the agreed-upon targets and, finally, sustainability of TB control.^2^ ^),(^ ^3^

These efforts (or overexertion) should consider the complexity of determinant factors of infection, including available health resources, educational level, income and occupation, population density, climate and living conditions in Brazil. Therefore, it is necessary to direct health interventions for TB control, which depends mainly on the evaluation of populations in their spatial context^7^ ^).(^ ^8^

In this sense, the continental dimensions of Brazil and the existence of regional inequalities imply the possibility of dissimilarities in levels of TB transmission, pointing to the importance of studies with different territorial profiles. This study aimed to analyze the distribution of TB cases in the state of Paraná, Southern Brazil, in the period from 2018 to 2021.

## METHODS

This was an ecological study on the health regions of Paraná, conducted using data from the Notifiable Heath Conditions Information System (Sistema de Informação de Agravos de Notificação - SINAN)/Ministry of Health and the Brazilian Institute of Geography and Statistics (Instituto Brasileiro de Geografia e Estatística - IBGE), accessed on May 17, 2022 via the Brazilian National Health System Information Technology Department (Departamento de Informática do Sistema Único de Saúde - DATASUS). This research is anchored in the REporting of studies Conducted using Observational Routinely-collected health Data (RECORD).^9^

The 27 Brazilian Federative Units (FUs) are distributed over five national macro-regions: North, Northeast, Southeast, South and Midwest. Paraná, according to IBGE estimates for 2021, is the most populous state in the South region, with 11,597,484 inhabitants, divided into four health macro-regions and 22 health regions, through which, by means of this decentralized administrative health organization, programs, actions and health services aimed at the population are implemented.^10^ ^),(^ ^11^

The study population was comprised of new cases of pulmonary TB reported on SINAN, obtained among the categories “new case”, “do not know” and “after death”, diagnosed in the period from 2018 to 2021. In order to define the target population, we used the historical landmark Brazil Free from Tuberculosis:^6^ ^)^ records made after the implementation of the program in 2018, and the most recent records available on SINAN, on the date when the authors had access to the system database, were included.

The variables “year of diagnosis” and “health region” where the notification was made were analyzed. Data were exported to Microsoft Excel 2016^®^ software and the absolute and relative frequencies were estimated. Detection rates (DR) were calculated year by year, based on the ratio of the number of cases over the estimated population, for the same period and health region, and the result multiplied by 100,000 inhabitants.

Regarding the rates that were calculated, the choropleth maps were built using the QGIS^®^ software, version 3.26.3, based on the shapefile with the boundaries of the regions, accessed on the Brazilian Open Data Portal website. The spatial distribution occurred through natural breaks, in which a color scale was assigned: the darkest shades corresponded to the highest rates, and the lightest shades, corresponded to the lowest rates.

Moreover, the data related to the rates were grouped into biennia. Based on the arithmetic mean of the years registered in the periods 2018-2019 and 2020-2021, the percentage change (PC) between both periods was estimated. This calculation was performed by subtracting the rates of the last biennium from the rates of the first biennium, followed by division of the value obtained by the first biennium, and the result multiplied by 100.

As this was a study that used data in the public domain, aggregated and without identification of the participants, its project was exempted from the approval of a Research Ethics Committee (REC). However, the norms and guidelines recommended by the National Health Council (Conselho Nacional de Saúde - CNS), Resolution No. 466 of December 12, 2012, were met.

## RESULTS

Between 2018 and 2021, 7,099 new cases of pulmonary TB were reported in the state of Paraná, distributed over the period as follows: 1,883 (26.5%; 16.6/100,000 inhabitants) in 2018; 1,915 (27.0%; 16.7/100,000 inhabitants) in 2019; 1,777 (25.0%; 15.4/100,000 inhabitants) in 2020; and 1,524 (21.5%; 13.1/100,000 inhabitants) in 2021 (data not shown in tables or figures).

**Figure 1:**
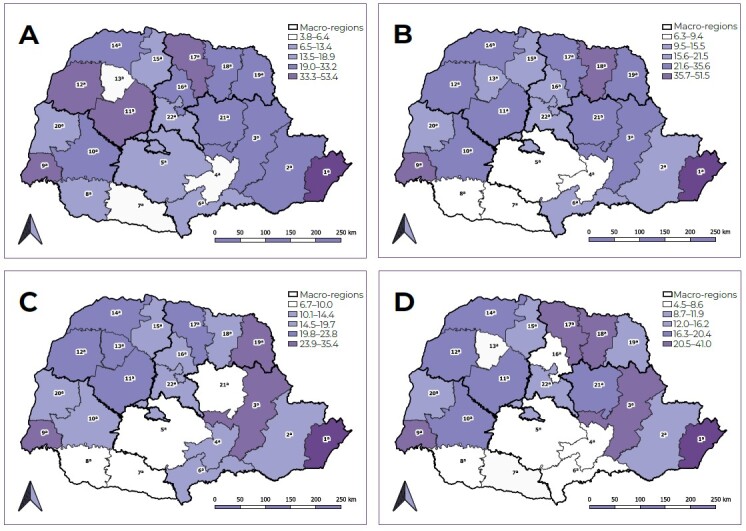
Spatial distribution of pulmonary tuberculosis detection rates (per 100,000 inhabitants) according to health regions, state of Paraná, Brazil, 2018 (A), 2019 (B), 2020 (C) and 2021 (D)

As for spatial distribution, the highest detection rates were observed mainly in the Northwest and North macro-regions of the state. It is worth highlighting that the health regions of Paranaguá and Foz do Iguaçu presented the highest rates for all the years analyzed, while the lowest values were found in Irati and Francisco Beltrão (Figure 1).

With regard to the percentage change of TB detection rates, it could be seen a decrease in all macro-regions, the highest was found in the West region (-22.8%). The health regions also showed variation, with a decrease in 18 of them, especially Foz do Iguaçu (-40.5%), Apucarana (-32.3%), União da Vitória (-29.9%) and Paranaguá (-27.2%), and an increase in four of them, with Cianorte (+53.6%) being the most significant (Table 1).

**Table 1:** Average detection rates of pulmonary tuberculosis (per 100,000 inhabitants) and percentage change, according to macro-regions and health regions, state of Paraná, Brazil, 2018-2019 and 2020-2021

Macro-regions and regions	2018-2019^a^	2020-2021^a^	Percentage change
East	16.0	13.9	-12.9
1^st^ Paranaguá	52.4	38.2	-27.2
2^nd^ Metropolitana	14.4	12.2	-15.2
3^rd^ Ponta Grossa	16.1	20.0	+23.8
4^th^ Irati	6.3	8.8	+39.2
5^th^ Guarapuava	9.8	7.8	-20.5
6^th^ União da Vitória	11.6	8.1	-29.9
21^st^ Telêmaco Borba	16.5	13.1	-20.5
West	16.6	12.8	-22.8
7^th^ Pato Branco	7.5	7.6	+1.5
8^th^ Francisco Beltrão	8.5	7.6	-10.6
9^th^ Foz do Iguaçu	34.4	20.5	-40.5
10^th^ Cascavel	15.9	13.7	-14.2
20^th^ Toledo	12.7	12.0	-5.6
Northwest	16.1	14.4	-10.1
11^th^ Campo Mourão	20.6	16.5	-20.0
12^th^ Umuarama	21.7	17.3	-20.4
13^th^ Cianorte	7.8	12.0	+53.6
14^th^ Paranavaí	18.0	17.7	-1.7
15^th^ Maringá	13.3	12.1	-8.8
North	19.3	16.6	-13.6
16^th^ Apucarana	16.0	10.8	-32.3
17^th^ Londrina	21.1	19.1	-9.4
18^th^ Cornélio Procópio	20.9	17.4	-16.6
19^th^ Jacarezinho	18.7	17.4	-6.8
22^nd^ Ivaiporã	13.9	12.2	-12.0
Paraná	16.7	14.3	-14.3

a) Arithmetic mean between years.

## DISCUSSION

The distribution of TB cases showed, among the years analyzed, a higher concentration in the North and Northwest macro-regions of Paraná, in addition to high rates in the health regions of Paranaguá and Foz do Iguaçu, and low rates in Irati and Francisco Beltrão. In 2020-2021, there was a decrease for almost all regions in the pandemic period, especially Foz do Iguaçu.

It is common knowledge that TB is a social disease of biological aspects, whose occurrence and transmission are connected to inequality and socioeconomic determinants.^8^ ^),(^ ^12^ ^),(^ ^13^ These characteristics are related to the contexts in which individuals live, given the high incidence found in vulnerable areas.^8^ ^),(^ ^14^

In Paraná, there was a predominance of cases in the North and Northwest macro-regions, close to the states of São Paulo and Mato Grosso do Sul, as well as in the border regions between the state and two South American countries - Argentina and Paraguay - and in the regions closest to the state’s coastal regions.

Border regions are considered marginalized, peripheral areas with deficit in socioeconomic integration, directly impacting the health of society.^15^ From the epidemiological point of view, it is important to consider the common characteristics of these regions, especially between twin cities, as in the health region of Foz do Iguaçu.^16^ ^)^

Therefore, the articulation of surveillance actions between countries is fundamental, given that for the transmission of the disease, there is no territorial limit.^16^ The intense cross-border flow toward Paraná reflects the search for more qualified health systems, overloading the services and negatively affecting the indicators of border health regions in the state.^17^

Furthermore, the highest detection may be associated with ecological factors. Territories with the lowest altitudes, such as the Paranaguá region, and the highest temperatures, such as in the North and Northwest macro-regions, report the highest rates of TB notification.^18^ ^)^ However, there is no consensus on this issue.

The results also revealed a decrease in the detection of the disease. Surveillance is a primary action of public health;^19^ ^),(^ ^20^ however, it is recognized that weak surveillance systems can lead to underreporting of TB.^21^ It is possible that this weakness may be accentuated in the context of COVID-19. Studies conducted in India and Sierra Leone showed a reduction of 63.3% and 70.0% in TB notifications, respectively.^22^ ^),(^ ^23^

The centralization of health actions and services in coping with the pandemic has put up barriers to the control of pre-existing conditions.^24^ ^),(^ ^25^ Therefore, it is understood that the interruption of health care activities has had significant impacts on the detection and management of TB.^26^ ^),(^ ^27^ In this scenario, it can be seen that the achievements in TB control were threatened in the pandemic scenario.

It is noteworthy that this research has limitations. The use of secondary data may be subject to errors in filling out and underreporting/sub-detection of cases, especially in a context aggravated by the pandemic. Another limitation of this study is related to detection rates calculated based on population estimates, which may not reflect the actual population size.

It can be concluded that this study showed a decrease in the detection of TB as a possible consequence of overload in health care and epidemiological surveillance, given the emergence of COVID-19. The analysis of spatial distribution pointed to a greater detection of TB cases in certain areas of the state of Paraná, suggesting relationships between these occurrences and ecological and socioeconomic aspects.
